# Kyasanur Forest Disease, India, 2011–2012

**DOI:** 10.3201/eid1902.120544

**Published:** 2013-02

**Authors:** Gudadappa S. Kasabi, Manoj V. Murhekar, Pragya D. Yadav, R. Raghunandan, S.K. Kiran, V.K. Sandhya, G.H. Channabasappa, Akhilesh C. Mishra, Devendra Tarachand Mourya, Sanjay M. Mehendale

**Affiliations:** Author affiliations: National Institute of Epidemiology, Chennai, India (G.S. Kasabi, M.V. Murhekar, S.M. Mehendale);; National Institute of Virology, Pune, India (P.D. Yadav, A.C. Mishra, D.T. Mourya);; Department of Health and Family Welfare of the Government of Karnataka in Shimoga District, Shimoga, India (G.S. Kasabi, R. Raghunandan, S.K. Kiran, G.H. Channabasappa);; Virus Diagnostic Laboratory, Shimoga, India (V.K. Sandhya)

**Keywords:** Kyasanur Forest disease, outbreak, vaccine efficacy, Shimoga, India, vector-borne infections, ticks, viruses

## Abstract

To determine the cause of the recent upsurge in Kyasanur Forest disease, we investigated the outbreak that occurred during December 2011–March 2012 in India. Male patients >14 years of age were most commonly affected. Although vaccination is the key strategy for preventing disease, vaccine for boosters was unavailable during 2011, which might be a reason for the increased cases.

Kyasanur Forest disease (KFD), a tick-borne viral disease, was first recognized in 1957 in Shimoga District, India, when an outbreak in monkeys in Kyasanur Forest was followed by an outbreak of hemorrhagic febrile illness in humans (*1*). KFD is unique to 5 districts (Shimoga, Chikkamagalore, Uttara Kannada, Dakshina Kannada, and Udupi) of Karnataka State and occurs as seasonal outbreaks during January–June (*2*–*4*).

Since 1990, vaccination campaigns using formalin-inactivated tissue-culture vaccine have been conducted in the districts to which KFD is endemic (Directorate of Health and Family Welfare Services, Government of Karnataka, Manual on Kyasanur Forest disease. 2005, unpub. data). Earlier studies showed vaccine efficacy of 79.3% with 1 dose and 93.5% with 2 doses (*5**,**6*). The vaccination program identifies villages reporting KFD activity (laboratory-confirmed cases in monkeys and/or humans, or infected ticks), and all villages within 5 km of the affected location are targeted for vaccination. Two doses are administered to persons 7–65 years of age at 1-month intervals. Because the immunity conferred by vaccination is short-lived, booster doses are administered at 6–9-month intervals consecutively for 5 years after the last reported KFD activity in the area (Directorate of Health and Family Welfare Services, Government of Karnataka, Manual on Kyasanur Forest disease. 2005, unpub. data). If KFD activity is reported where vaccination has been administered during pretransmission seasons, additional vaccination campaigns are conducted.

Thirthahalli Taluka in the Shimoga District (Figure 1), where vaccination campaigns were ongoing, reported 0 cases of KFD during 2007–2010. A vaccination campaign was conducted in the area during October 2010. Because 11 cases were reported from the Thirthahalli Taluka in March 2011, vaccination campaigns were conducted during April–May 2011; however, no booster doses were administered in the affected areas during October–November 2011 because the vaccine was not available. Suspected KFD cases were reported in the area again in December 2011. We investigated this cluster to 1) confirm the etiology, 2) identify risk factors, and 3) propose recommendations for control.

**Figure 1:**
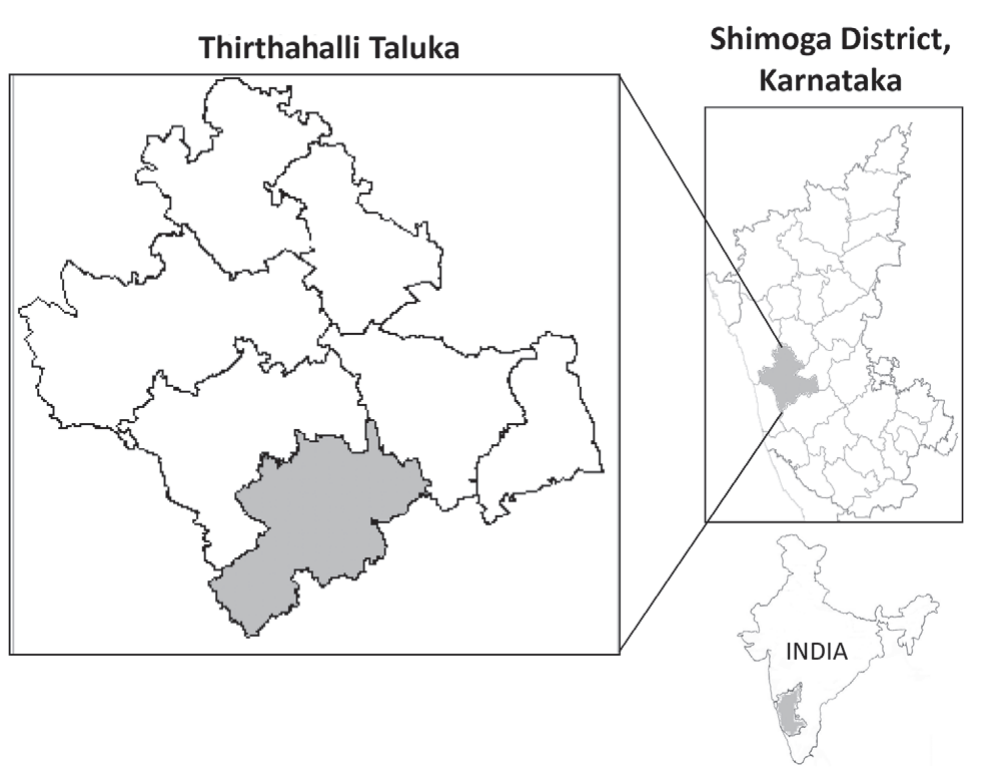
Location of Thirthahalli Taluka, Shimoga District, Karnataka State, India.

## The Study

We defined a suspected KFD case as sudden onset of fever, headache, and myalgia among residents of Shimoga during December 2011–March 2012 (Directorate of Health and Family Welfare Services, Government of Karnataka, Manual on Kyasanur Forest disease, 2005, unpub. data). Health workers conducted door-to-door searches to identify suspected case-patients within 5 km of villages that reported monkey deaths or laboratory-confirmed KFD cases in humans since December 2011. We established stimulated passive surveillance in health facilities in the district to identify suspected case-patients. Health workers collected information about sociodemographic profile, date of onset, and place of residence from all suspected case-patients. We recorded clinical history and vaccination details of laboratory-confirmed case-patients. We analyzed the data to describe the disease by time, place, and person. The investigation was exempted from ethical committee clearance because it was part of the state-level public health response to the outbreak.

Blood specimens were collected from all suspected case-patients. We tested for KFD virus by using nested reverse transcription PCR (RT-PCR) and Taqman-based RT-PCR at the National Institute of Virology (Pune, India) (*7*) and/or intracerebral injection of the serum into suckling mice at the Virus Diagnostic Laboratory, Shimoga (Technical Appendix).

We conducted a matched case–control study to identify risk factors for the illness. Persons with laboratory-confirmed infection who were admitted to health facilities were considered case-patients, and healthy persons were used as controls. We recruited 2 controls per case-patient (total 51 cases, 102 controls). Case-patients and controls were matched with age group (±5 years), sex, and locality. We interviewed participants to collect information about any recent exposure to the forest and number of doses of KFD vaccine received in 2011. We conducted conditional logistic regression analysis by using Epi Info software (Centers for Disease Control and Prevention, Atlanta, GA, USA) to identify risk factors. All risk factors evaluated were included in the logistic regression model.

**Figure 2:**
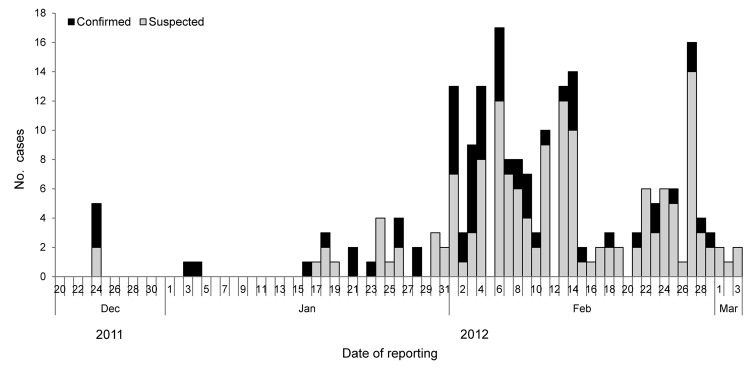
Distribution of suspected and confirmed Kyasanur Forest disease cases, Shimoga District, Karnataka State, India, December 2011–March 2012.

During December 2011–March 2012, we identified 215 suspected case-patients from 80 villages (total population 22,201) in Shimoga (attack rate 9.7 cases/1,000 persons). Of these, 61 (28%) cases were laboratory confirmed (57 by RT-PCR; 4 by suckling mice intracerebral inoculation). Most (92%) laboratory-confirmed case-patients were >14 years of age, and 70% were male (Table 1). The cases began occurring in the last week of December 2011, peaked during the first 2 weeks of February, and then declined gradually (Figure 2). Of the 215 suspected cases, 166 (77%) occurred in 4 primary health center areas of Thirthahalli Taluka.

**Table 1:** Age and sex distribution of suspected and laboratory-confirmed case-patients during Kyasanur Forest disease outbreak, Shimoga District, Karnataka State, India, December 2011–March 2012*

Characteristic	Population	Suspected			Laboratory-confirmed	
		No. case-patients	Attack rate*		No. case-patients	Attack rate*
Age group, y						
<14	7,193	32	4		5	1
15–29	6,349	51	8		16	3
30–44	4,440	70	16		22	5
45–59	2,642	48	18		13	5
>60	1,577	14	9		5	3
Sex						
M	11,194	136	12		43	4
F	11,007	79	7		18	2
Total	22,201	215	10		61	3

*Per 1,000 population.

Besides fever and myalgia, common clinical manifestations among the 61 laboratory-confirmed case-patients included bleeding (38 [63%] persons), vomiting (28 [46%]), and abdominal pain (26 [42%]). The hemorrhagic manifestations included conjunctival congestion (30 [49%]), hematemesis (5 [8%]), epistaxis (1 [2%]), hematuria (1 [2%]), and rectal bleeding (1 [2%]). One patient died (case-fatality rate 0.5%). Of the 61 laboratory-confirmed case-patients, 20 (33%) had received 2 doses of KFD vaccine, and 2 (3%) received 1 dose; 39 (64%) did not receive any vaccination during April–May 2011. Twelve case-patients were housewives or students; the rest reported multiple occupations requiring frequent visits to the forest, such as cultivator, dry leaf gatherer, agriculture laborer, and cattle grazer.

Behavioral factors, such as handling cattle (adjusted odds ratio [aOR] 5.1, 95% CI 1.3–20.4) and frequent visits to forest for livelihood (aOR 4.8, 95% CI 1.2–20.3) and piles of dry leaves within the compounds of the house (aOR 4.1, 95% CI 1.3–12.3) were independently associated with illness. Of the 51 case-patients, 20 had received 2 doses of vaccine and 2 had received 1 dose. The odds of developing illness did not differ significantly for nonvaccinated case-patients and case-patients who received 2 doses (Table 2).

**Table 2:** Univariate and multivariate analysis of risk factors associated with Kyasanur Forest disease outbreak, Shimoga District, Karnataka State, India, December 2011–March 2012

Variable	No. (%) case-patients, n = 51	No. (%) controls, n = 102	Odds ratio (95% CI)	
			Matched	Adjusted
Handled cattle in last week	47 (92)	72 (71)	5.4 (1.6–18.2)	**5.1 (1.3–20.4)**
Went to forest in last week	44 (86)	72 (71)	4.2 (1.2–14.3)	**4.7 (1.1–20.3)**
Had pile of leaves within the compound	39 (76)	58 (57)	3.2 (1.3–7.9)	**4.0 (1.3–12.3)**
Had cattle shed in household	48 (94)	89 (87)	3.3 (0.7–16.3)	3.7 (0.5–25.9)
Received 2 doses of vaccine in 2011	20 (39)	42 (41)	0.7 (0.2–2.9)	2.4 (0.4–15)
Used tick repellent before going to forest	8 (16)	18 (18)	0.8 (0.3–2.4)	1.1 (0.3–3.8)

*Boldface indicates significance.

## Conclusions

Vaccination is the key strategy for preventing KFD in Karnataka. However, during 2011, a booster vaccination campaign was not conducted in the district because of vaccine unavailability, which might be a reason for the upsurge of KFD cases during 2012. Two doses of the vaccine given during April–May 2011 did not confer adequate protection against the disease during December 2011–March 2012, suggesting the possibility of short-lived immunity conferred by 2 doses of vaccine and the need for periodic boosters.

In the affected areas, local villagers stay in and around the forest area, frequently visit the forest for their livelihood, and get infected through tick bites. We identified certain risk factors for the illness, including frequent visits to the forest, handling of cattle, and piles of dry leaves within the compounds. The higher attack rates for male case-patients aged >14 years during this outbreak are consistent with their frequent exposure to the forest. Health authorities advise use of tick repellent; however, it was infrequently used in the area. Educating the community to wear long-sleeved clothing might help reduce exposure to ticks.

Although the transmission cycle of KFD virus is well documented, its control remains challenging. Measures to minimize the human–tick interface are less likely to succeed considering the forest ecosystem and the dependence of local villagers on it. Control of ticks in the forest is far from easy, but health authorities need to continue educating villagers about using tick repellent before visiting the forest, especially during spring and summer, and ensure distribution of tick repellents to them. Health authorities must ensure that vaccination campaigns are initiated on time and completed before November every year. More epidemiologic studies are needed to evaluate the long-term protection offered by booster doses of vaccine. Molecular studies also are needed to understand the phylogenetic relationships of the past and contemporary strains of the virus and to identify possible sources and origins of outbreak strains.

Technical Appendix Details of suckling mice intracerebral inoculation with serum from patients with suspected Kyasanur Forest virus.
