# Supplementary material for: Kyasanur Forest Disease, India, 2011–2012
Source: Emerg Infect Dis. 2013 Feb;19(2):278–81. doi: 10.3201/eid1902.120544 (PMC3559039; doi:10.3201/eid1902.120544)
Supplement: Technical Appendix — Details of suckling mice intracerebral inoculation with serum from patients with suspected Kyasanur Forest virus. [file 12-0544-Techapp-s1.pdf]

Article DOI: http://dx.doi.org/10.3201/eid1902.120544

Kyasanur Forest Disease, India, 2011–2012

Technical Appendix

Details of suckling mice intracerebral inoculation: 0.02 mL of patient's serum was inoculated into 3-day-old Swiss albino mice by intracerebral route. The criteria for the positive test result included development of drowsiness, feathering, spasticity of legs, and paralysis. The brains of sick mice were then harvested and diluted with 0.75% bovine albumin in phosphate saline and intracerebrally inoculated into a group of 6 adult mice and observed for 21 days for sickness. Presence of above-mentioned sickness confirmed KFD infection. The aliquots of the serum samples from patients found positive by virus isolation were also confirmed at NIV, Pune, by KFD-specific RT-PCR.
